# Carbon dioxide fixation by Calvin-Cycle enzymes improves ethanol yield in yeast

**DOI:** 10.1186/1754-6834-6-125

**Published:** 2013-08-29

**Authors:** Víctor Guadalupe-Medina, H Wouter Wisselink, Marijke AH Luttik, Erik de Hulster, Jean-Marc Daran, Jack T Pronk, Antonius JA van Maris

**Affiliations:** 1 Department of Biotechnology, Delft University of Technology, Julianalaan 67, 2628, BC Delft, The Netherlands; 2 Kluyver Centre for Genomics of Industrial Fermentation, P.O. Box 5057, 2600, GA Delft, The Netherlands

**Keywords:** Metabolic engineering, Synthetic biology, Rubisco, Ribulose-1,5-bisphosphate carboxylase, Phosphoribulokinase, NADH re-oxidation, Carbon dioxide fixation, *Saccharomyces cerevisiae*, Glycerol, Bioethanol

## Abstract

**Background:**

Redox-cofactor balancing constrains product yields in anaerobic fermentation processes. This challenge is exemplified by the formation of glycerol as major by-product in yeast-based bioethanol production, which is a direct consequence of the need to reoxidize excess NADH and causes a loss of conversion efficiency. Enabling the use of CO_2_ as electron acceptor for NADH oxidation in heterotrophic microorganisms would increase product yields in industrial biotechnology.

**Results:**

A hitherto unexplored strategy to address this redox challenge is the functional expression in yeast of enzymes from autotrophs, thereby enabling the use of CO_2_ as electron acceptor for NADH reoxidation. Functional expression of the Calvin cycle enzymes phosphoribulokinase (PRK) and ribulose-1,5-bisphosphate carboxylase (Rubisco) in *Saccharomyces cerevisiae* led to a 90% reduction of the by-product glycerol and a 10% increase in ethanol production in sugar-limited chemostat cultures on a mixture of glucose and galactose. Co-expression of the *Escherichia coli* chaperones GroEL and GroES was key to successful expression of CbbM, a form-II Rubisco from the chemolithoautotrophic bacterium *Thiobacillus denitrificans* in yeast.

**Conclusions:**

Our results demonstrate functional expression of Rubisco in a heterotrophic eukaryote and demonstrate how incorporation of CO_2_ as a co-substrate in metabolic engineering of heterotrophic industrial microorganisms can be used to improve product yields. Rapid advances in molecular biology should allow for rapid insertion of this 4-gene expression cassette in industrial yeast strains to improve production, not only of 1st and 2nd generation ethanol production, but also of other renewable fuels or chemicals.

## Background

The yeast *Saccharomyces cerevisiae* is not only used for the large-scale production of fuel ethanol [1], but also for industrial production of a broad and rapidly expanding range of other chemical compounds from renewable carbohydrate feedstocks [2,3]. In anaerobic, ethanol-producing cultures of *S. cerevisiae*, excess NADH generated from biosynthetic reactions, such as NAD^+^-dependent oxidative decarboxylations involved in synthesis of the precursors acetyl-CoA and 2-oxoglutarate, is reoxidized by reducing part of the sugar substrate to glycerol [4]. In growing anaerobic yeast cultures, glycerol production typically accounts for 4-10% of the total sugar consumption and therefore has a significant impact on ethanol yields and process economy in both 1st and 2nd generation large-scale bioethanol production [5,6].

**Figure 1:**
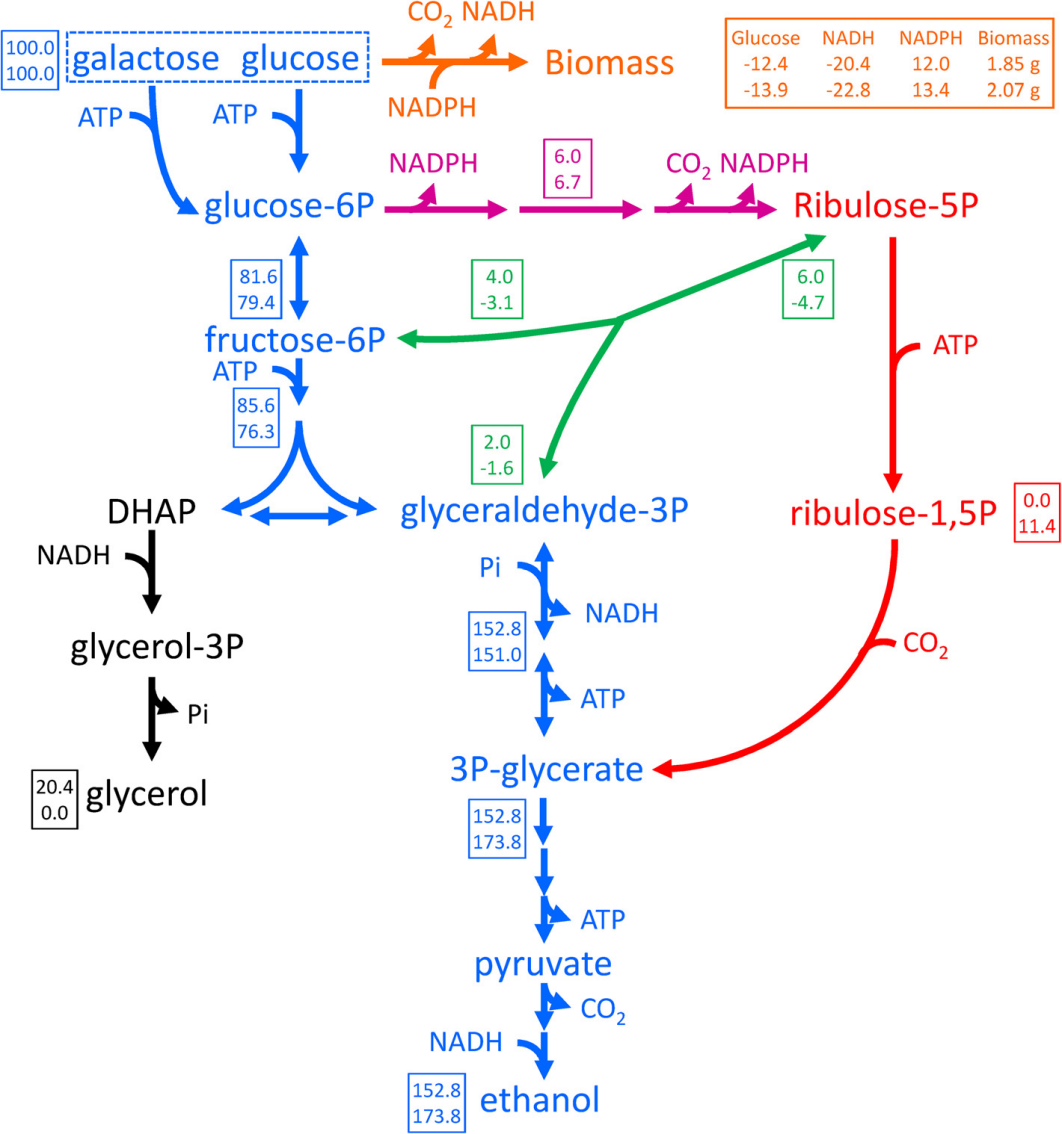
**Schematic representation of central carbon metabolism and the introduced Calvin-cycle enzymes in *Saccharomyces cerevisiae*.** Orange: Formation of biomass and NADH from glucose and NADPH. Stoichiometries are according to Verduyn *et al.*[7]; Blue: Redox-neutral, ATP-yielding alcoholic fermentation of glucose and galactose via the Embden-Meyerhof-Parnas glycolysis and Leloir pathways, respectively; Magenta: NADPH generation via the oxidative part of the pentose-phosphate pathway; Green: rearrangement of sugar-phosphate carbon skeletons via the non-oxidative pentose-phosphate pathway; Black: NADH oxidation by formation of glycerol through glycerol-3-phosphate dehydrogenase and glycerol-3-phosphatase; Red: heterologously expressed Calvin-cycle enzymes phosphoribulokinase and Rubisco. Numbers in boxes represents the distribution of carbon along the different pathways (in mmol) normalized for a combined glucose and galactose uptake of 100 mmol for a wild-type, glycerol-producing reference strain (top) and for a scenario in which the alternative pathways via the Calvin cycle enzymes completely replace glycerol formation as the mechanism for reoxidizing NADH formed in biosynthetic reactions (bottom). In the scenario with the Calvin cycle enzymes, ribulose-5-phosphate was assumed to be preferentially derived from the oxidative reactions of the pentose phosphate pathway. Once the generation of NADPH from these reactions matched the requirement for NADPH in biosynthesis, further ribulose-5-phosphate was derived from glycolytic intermediates via the non-oxidative pentose-phosphate pathway rearrangement reactions. The biomass yield on ATP was assumed to be identical for both scenarios.

Using CO_2_ as electron acceptor for the reoxidation of NADH would be a highly attractive metabolic engineering strategy, in particular when CO_2_ reduction can be coupled to the formation of the product of interest. Functional expression of the Calvin cycle enzymes phosphoribulokinase (PRK) and ribulose-1,5-bisphosphate carboxylase (Rubisco) in *S. cerevisiae* should enable the coupling of CO_2_, a major product of alcoholic fermentation, to ribulose-5-phosphate, a normal intermediate of the *S. cerevisiae* pentose-phosphate pathway (Figure 1). The resulting two molecules of 3-phosphoglycerate can subsequently be converted to 2 molecules each of ethanol and CO_2_, with the concomitant net oxidation of 2 molecules of NADH to NAD^+^ (Figure 1). When ribulose-5-phosphate is formed via the oxidative pentose-phosphate pathway (Figure 1), this route results in a transhydrogenase-type conversion of redox cofactors (NADP^+^ + NADH → NADPH + NAD^+^). Since the total amount of NADPH required in biosynthesis is smaller than the amount of NADH generated [7], such a transhydrogenase-like activity cannot fully replace glycerol formation as a mechanism for reoxidizing biosynthetic NADH. However, no such constraint exists when ribulose-5-phosphate is formed from intermediates of glycolysis via the rearrangement reactions of the non-oxidative pentose-phosphate pathway (Figure 1). A theoretical analysis shows that complete replacement of glycerol production with CO_2_ incorporation through PRK and Rubisco can increase the ethanol yield of sugar by as much as 14% (Figure 1).

The PRK gene from *Spinacia oleracea*[8] has previously been expressed in the yeast *Pichia pastoris*[9] and is therefore an interesting candidate for heterologous expression in *S. cerevisiae*. For Rubisco, a key enzyme in the Calvin cycle for autotrophic carbon fixation, three catalytically active forms have been described [10,11]. Prokaryotic form-II Rubisco’s are encoded by single structural genes and several have been heterologously expressed in *E. coli*[12,13]. Functional expression of form-II Rubisco’s in *E. coli* was shown to be strongly stimulated by the *E. coli* protein-folding chaperones GroEL and GroES [14] and expression of *Hydrogenovibrio marinus* Rubisco in *E. coli* was further stimulated by co-expression of the CbbO and CbbQ chaperones of the donor organism [15]. Very recently the structure of GroEL/GroES encapsulating Rubisco was visualized by cryo-electron microscopy [16]. Eukaryotes such as *S. cerevisiae* harbour a chaperone couple (Hsp60/Hsp10) that structurally and functionally resemble GroEL/GroES. However, these proteins are located in the mitochondria, whereas a role in Rubisco expression would require their activity in the cytosol.

In this study we investigated how to achieve functional expression of PRK and Rubisco in yeast. In view of the envisioned benefit of being encoded by single structural genes, a prokarytic form-II Rubisco gene was expressed in *S. cerevisiae* in combination with the PRK gene from *Spinacia oleracea*. Both the promoters and coding regions for genes required for glycerol formation were left unchanged compared to the reference strain. Subsequently, the impact of the resulting CO_2_ incorporation on product formation was studied, with special emphasis on the yields of ethanol and the undesired by-product glycerol.

## Results and discussion

### Chaperone-mediated functional expression of Rubisco in *Saccharomyces cerevisiae*

**Figure 2:**
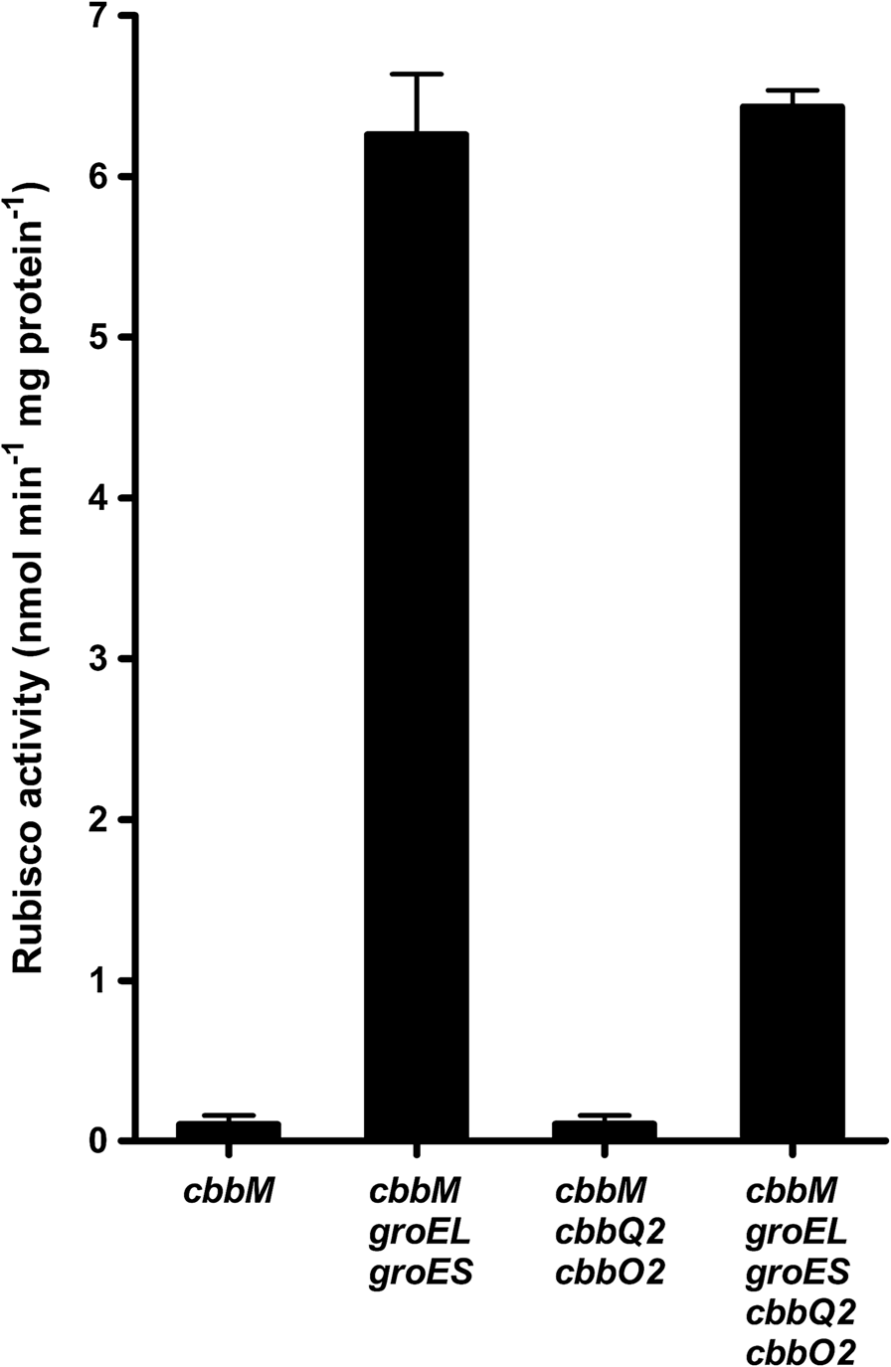
**Rubisco enzymatic activity in *S. cerevisiae *strains expressing different synthetic constructs.** Specific ribulose-1,5-bisphosphate carboxylase (Rubisco) activity in cell extracts of *S. cerevisiae* expressing Rubisco form II CbbM from *T. denitrificans*, either alone (IMC033) or in combination with the *E. coli* chaperones GroEL/GroES [18] (IMC035), the *T. denitrificans* chaperones CbbO2/CbbQ2 [20] (IMC034) or all four chaperones (IMC014). Heterologously expressed genes were codon optimised for expression in yeast and expressed from a single centromeric vector. Biomass samples were taken from anaerobic batch cultures on synthetic media (pH 5.0, 30°C), sparged with nitrogen and containing 20 g l^-1^ glucose as carbon source. Rubisco activities, measured as ^14^CO_2_-fixation in cell extracts, in a wild-type reference strain and in *S. cerevisiae* strains expressing *cbbM* and *cbbM*-*cbbQ2*-*cbbO2* were below the detection limit of the enzyme assay (0.2 nmol CO_2_ min^-1^ mg protein^-1^).

To study a possible requirement of heterologous chaperones for expression of Rubisco in *S. cerevisiae*, the form-II Rubisco-encoding *cbbM* gene from *T. denitrificans*[17] was codon-optimised for expression in *S. cerevisiae* and expressed from a centromeric vector, both alone and in combination with expression cassettes for the codon-optimised *E. coli groEL*/*groES*[18] and/or *T. denitrificans cbbO2/cbbQ2* genes [19,20]. Functional expression of *T. denitrificans* Rubisco in *S. cerevisiae*, as indicated by ribulose-1,5-bisphosphate-dependent ^14^CO_2_ fixation by yeast cell extracts, was only observed upon co-expression of *E. coli* GroEL/GroES (Figure 2). Co-expression of CbbO2/CbbQ2 did not result in a further increase of Rubisco activity (Figure 2). Co-expression of bacterial chaperones has previously been shown to improve heterologous protein expression in *Pichia pastoris* and insect cells [21,22]. The positive effect of GroEL/GroES on Rubisco expression in *S. cerevisiae* demonstrates the potential value of co-expression of heterologous chaperones for metabolic pathway engineering that requires expression of prokaryotic enzymes in the cytosol of eukaryotes.

### Functional expression of phosphoribulokinase in *Saccharomyces cerevisiae*

The *Spinacia oleracea* phosphoribulokinase (PRK) gene [8], which has previously been expressed in the yeast *Pichia pastoris*[9], was integrated together with *E. coli groEL*/*groES* and *T. denitrificans cbbO2/cbbQ2* into the *S. cerevisiae* genome at the *CAN1* locus, under control of the galactose-inducible *GAL1* promotor. This resulted in high PRK activities (approximately 15 μmol mg protein^-1^ min^-1^) in cell extracts of *S. cerevisiae* strain IMU033 taken from carbon-limited chemostat cultures on a mixture of glucose and galactose (Table 1). Although relatively high background activities were measured in the reference strain without PRK (IMU032), this activity does not contribute to pathway activity (see below). We therefore assumed that the background activity observed in the reference strain was caused by an impurity in one of the chemicals used in the assay and did not reflect formation of ribulose-1,5-bisphosphate. The engineered strain IMU033, which additionally carried the centromeric expression cassette for *T. denitrificans* Rubisco, was used to quantitatively analyse the physiological impacts of the expression of Rubisco and PRK.

**Table 1:** **Physiological analysis of *S. cerevisiae *IMU033 expressing *PRK *and Rubisco and the isogenic reference strain IMU032 in anaerobic chemostat cultures, grown at a dilution rate of 0.05 h**^**-1 **^**on a synthetic medium (pH 5) supplemented with 12.5 g l**^**-1 **^**glucose and 12.5 g l**^**-1 **^**galactose as carbon sources**^**#**^

	**IMU032**		**IMU033**	
	**(Reference strain)**		**(Expressing PRK and Rubisco)**	
CO_2_ in inlet gas (%)	0	10	0	10
CO_2_ in outlet gas (%)	0.89 ± 0.03	10.8 ± 0.0	1.02 ± 0.00	10.8 ± 0.1
Phosphoribulokinase (μmol mg protein^-1^ min^-1^)	0.58 ± 0.09	0.51 ± 0.12	14.4 ± 1.5	15.2 ± 1.0
Rubisco (nmol mg protein^-1^ min^-1^)	< 0.2*	< 0.2	4.59 ± 0.30	2.67 ± 0.28
Biomass yield on sugar (g g^-1^)	0.083 ± 0.000^a^	0.084 ± 0.000^b^	0.093 ± 0.001^a^	0.095 ± 0.000^b^
Ethanol yield on sugar (mol mol^-1^)	1.56 ± 0.03^c^	1.56 ± 0.02^d^	1.73 ± 0.02^c^	1.73 ± 0.01^d^
Glycerol yield on sugar (mol mol^-1^)	0.14 ± 0.00^e^	0.12 ± 0.00^f^	0.04 ± 0.00^e, g^	0.01 ± 0.00^f, g^

^#^Results are represented as average ± mean deviations of data from independent duplicate chemostat experiments. Data pairs labelled with the same subscripts (^a,a^, ^b,b^, etc.) are considered statistically different in a standard *t*-test (p <0.02).

*Detection limit of enzyme activity assay.

### Carbon dioxide as electron acceptor in anaerobic chemostat cultures of *Saccharomyces cerevisiae*

Quantitative physiological analysis is facilitated by the constant and highly reproducible process conditions in steady-state chemostat cultures [23,24]. Therefore, ethanol and glycerol yields of PRK- and Rubisco-expressing *S. cerevisiae* were compared to those of an isogenic reference strain in anaerobic, sugar-limited chemostats on a mixture of 12.5 g l^-1^ glucose and 12.5 g l^-1^ galactose. In nitrogen-sparged cultures, the glycerol yield on sugar in the strain expressing both Calvin-cycle enzymes was 68% lower than in the reference strain, while ethanol and biomass yields on sugar were 11% and 12% higher, respectively (Table 1). To investigate whether the low affinity of *T. denitrificans* form-II Rubisco for CO_2_ (K_CO2_ = 0.26 mM [17]) limited its *in vivo* activity in the nitrogen-sparged cultures, additional chemostats were sparged with a 10%/90% blend of CO_2_ and N_2_. Indeed, this CO_2_ supplementation resulted in a further decrease of the glycerol yield to a value below 10% of that of the reference strain (Table 1). Co-expression of Rubisco and chaperones without co-expression of PRK (strain IMC014) did not result in decreased glycerol yield (0.13 mol mol^-1^) compared to the reference strain IMU032 (0.12 mol mol^-1^) in carbon-limited chemostat cultures supplemented with CO_2_. This observation confirmed that expression of a heterologous phosphoribulokinase (PRK) gene is required for *in vivo* carbon fixation via Rubisco in yeast.

### Carbon dioxide as electron acceptor in anaerobic batch fermentations

**Figure 3:**
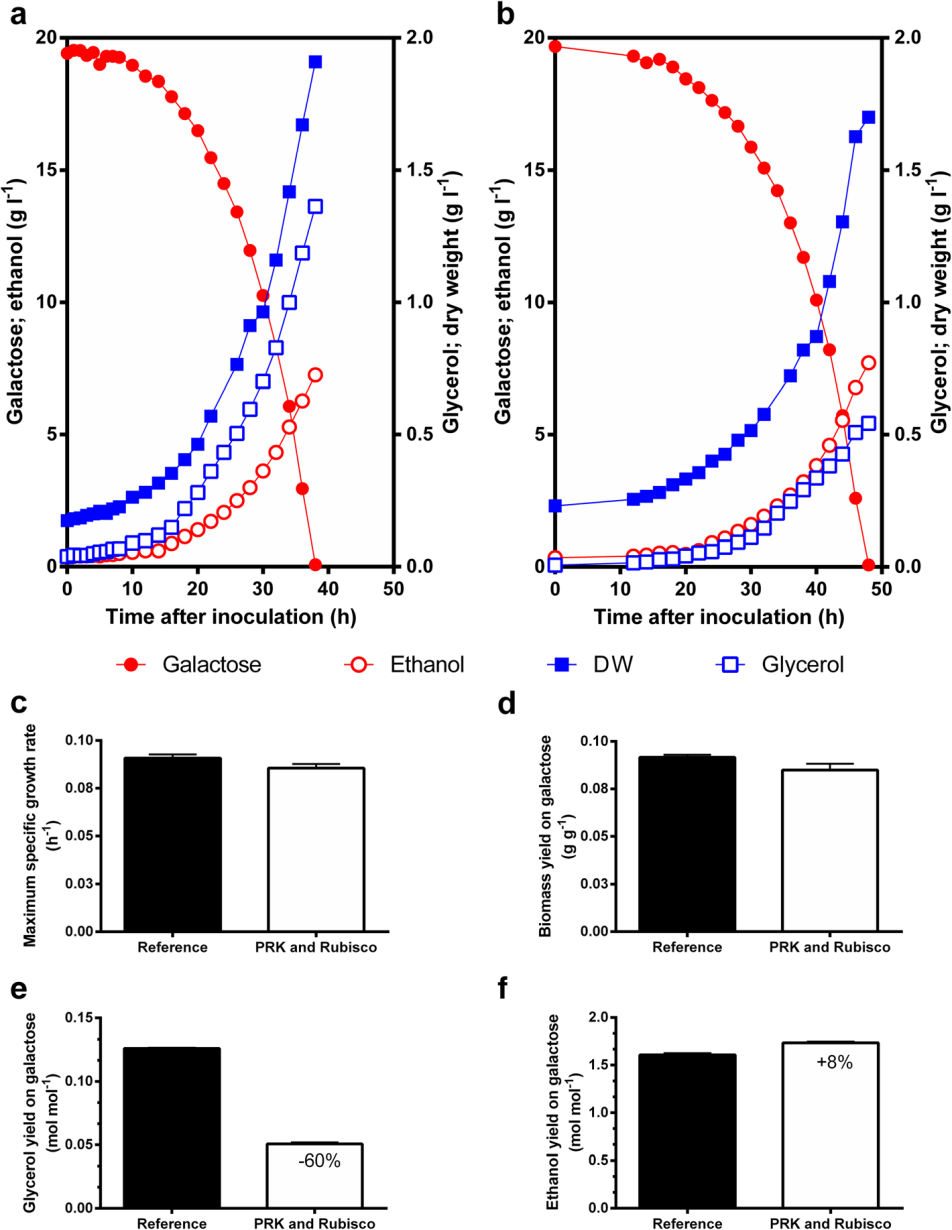
**Physiological impact of expression of Calvin cycle enzymes on growth, substrate consumption and product formation in galactose-grown anaerobic batch cultures of *S. cerevisiae*. a**: growth curves of isogenic reference strain *S. cerevisiae* IMU032, **b**: growth curves of *S. cerevisiae* IMU033 expressing PRK and Rubisco. Growth conditions: T = 30°C, pH 5.0, 10% CO_2_ in inlet gas. Each graph represents values for one of two independent replicate experiments, whose growth kinetic parameters differed by less than 5%. **c-f**: Calculated parameters: Maximum specific growth rate **(c)**, biomass yield **(d)**, glycerol yield **(e)**, and ethanol yield **(f)** on galactose of the isogenic *S. cerevisiae* reference (black bars) and strain expressing PRK and Rubisco (white bars). Results are represented as average ± mean deviations of data from independent duplicate cultures. Values inside the white bars represent statistically significant differences in a standard *t*-test (p value < 0.02) relative to the reference strain.

Since industrial-scale ethanol production is routinely performed in batch fermentations [25], the impact of the expression of PRK and Rubisco was also investigated in anaerobic, CO_2_-supplemented batch cultures (Figure 3). Galactose was used as the carbon source for these experiments to enable efficient expression of PRK from the *GAL1* promoter. Despite an almost 10 h difference in the lag phase, the specific growth rates of the engineered and reference strains on galactose in these anaerobic cultures were not significantly different (Figure 3) and in good agreement with values reported elsewhere for this yeast strain family [26]. Consistent with the observations in chemostat cultures, expression of the two Calvin cycle enzymes reduced glycerol formation in the batch cultures by 60% and increased the ethanol yield on galactose by 8% (Figure 3e-f). However, the biomass yield on sugar, which in the chemostat cultures increased according to the predictions, did not increase in the anaerobic batch cultures on galactose. These observations together might indicate that under the galactose excess conditions used for these batch cultivations increased expression levels of PRK result in a small metabolic burden, but still result in an overall positive effect on the ethanol yield.

## Conclusions

This study provides a compelling proof of principle for the replacement of glycerol formation as the predominant redox sink in anaerobic yeast metabolism by PRK- and Rubisco-mediated incorporation of CO_2_ into yeast central carbon metabolism. The loss of sugar feedstock due to glycerol production in industrial bioethanol processes has been estimated at 4% of the consumed sugar [5]. If expression of PRK and Rubisco in industrial yeast strains were to completely eliminate this loss, this could enable an additional production of 5 billion liters of ethanol from the amount of sugar used for the 2011 global ethanol production of 110 billion liters [1]. Use of CO_2_ as an external electron acceptor offers important advantages over previously proposed strategies for reducing glycerol production in yeast-based bioethanol production. Optimizing the redox cofactor specificity of nitrogen assimilation in *S. cerevisiae*[5] only enables a partial reduction of glycerol production and its impact further depends on the nitrogen sources present in industrial feedstocks. Similarly, a metabolic engineering strategy that enables NADH-dependent reduction of acetic acid [6] to ethanol is dependent on the presence of acetic acid in industrial feedstocks. Further optimization of PRK and Rubisco gene expression and regulation in *S. cerevisiae* should enable the design and construction of DNA cassettes that can be easily introduced in the genomes of industrial yeast strains. This should include the replacement of the *GAL1*-promotor, that was used for the expression of PRK in this study, by a promotor that is compatible with fast growth at high glucose concentrations and that further balances the expression of PRK and Rubisco. Since ribulose-5-phosphate is also an intermediate in pentose metabolism by engineered *S. cerevisiae* strains [27], this approach should also be readily applicable to the yeast-based conversion of lignocellulosic hydrolysates. The observed stimulatory effect of CO_2_ on the engineered strains will not hinder application of this concept in industrial bioethanol production, since large-scale processes for bioethanol production are characteristically CO_2_ saturated.

Our results illustrate how metabolic engineering strategies based on the functional integration of extensively studied reactions in the central carbon metabolism of distantly related organisms enables the optimization of product yields in industrial biotechnology. Although the present study focuses on ethanol production by yeast, functional integration of autotrophic carbon-fixing enzymes in the metabolic networks of industrial microorganisms should also enable optimization of yields of other existing and novel products whose synthesis results in a net positive ATP yield.

## Methods

### Construction of expression modules

Phosphoribulokinase (*PRK*) cDNA from *Spinacia oleracea* (spinach) [9] (accession number: X07654.1) was PCR-amplified using Phusion Hot-Start Polymerase (Finnzymes, Landsmeer, the Netherlands) and the oligonucleotides XbaI_prk_FW2 and RV1_XhoI_prk (Table 2), and was ligated in pCR®-Blunt II-TOPO® (Life Technologies Europe BV, Bleiswijk, the Netherlands). After restriction by XbaI and XhoI, the PRK-containing fragment was ligated into pTEF424 [28]. The *TEF1*p was later replaced by *GAL1*p from plasmid pSH47 [29] by XbaI and SacI restriction/ligation, creating plasmid pUDE046 (Table 3).

**Table 2:** Oligonucleotides used in this study

**Number**	**Name**	**Sequence (5′ to 3′)**	**Purpose**
	**Cloning**		
1	XbaI_prk_FW2	TGACATCTAGATGTCACAACAACAAACAATTG	Cloning of PRK into pUDE046.
2	RV1 XhoI prk	TGACATCTAGATGTCACAACAACAAACAATTG	Cloning of PRK into pUDE046.
	**Primers used for *in vivo *plasmid assembly**		
3	HR-cbbM-FW-65	TTGTAAAACGACGGCCAGTGAGCGCGCGTAATACGACTCACTATAGGGCGAATTGGGTACAGCTGGAGCTCAGTTTATCATTATC	Rubisco *cbbM* cassette for plasmids pUDC075, pUDC099, and pUDC100.
4	HR-cbbM-RV-65	GGAATCTGTGTAGTATGCCTGGAATGTCTGCCGTGCCATAGCCATGTATGCTGATATGTCGGTACCGGCCGCAAATTAAAG	Rubisco *cbbM* cassette for plasmids pUDC075, pUDC099, and pUDC100
5	linker-cbbO2-pRS416	ATCACTCTTACCAGGCTAGGACGACCCTACTCATGTATTGAGATCGACGAGATTTCTAGGCCAGCTTTTGTTCCCTTTAGTGAGGGTTAATTGCGCGCTTGGCGTAATCATGGTCATAGC	Linker fragment for assembly of plasmid pUDC099.
6	linker-cbbM-GroEL	GACATATCAGCATACATGGCTATGGCACGGCAGACATTCCAGGCATACTACACAGATTCCATCACTCTTACCAGGCTAGGACGACCCTACTCATGTATTGAGATCGACGAGATTTCTAGG	Linker fragment for assembly of plasmid pUDC100.
	**Primers used for *in vivo *integration assembly**		
7	FW pTDH3- HR-CAN1up	GTTGGATCCAGTTTTTAATCTGTCGTCAATCGAAAGTTTATTTCAGAGTTCTTCAGACTTCTTAACTCCTGTAAAAACAAAAAAAAAAAAAGGCATAGCAAGCTGGAGCTCAGTTTATC	1st cloning expression cassette linker fragment between *CAN1* upstream and PRK expression cassette (IMI229), and CAN1up-linker and *KlLEU2* expression cassette (IMI232).
8	RV linker-iHR2B	AGATATACTGCAAAGTCCGGAGCAACAGTCGTATAACTCGAGCAGCCCTCTACTTTGTTGTTGCGCTAAGAGAATGGACC	1st cloning fragment: linker fragment between CAN1up-linker and PRK expression cassette (IMI229).
9	RV linker-iHR6	GCTATGACCATGATTACGCCAAGCGCGCAATTAACCCTCACTAAAGGGAACAAAAGCTGGTTGCGCTAAGAGAATGGACC	1st cloning fragment: linker fragment between CAN1up-linker and *KlLEU2* expression cassette (IMI232).
10	FW pGAL1-prk HR2B	CAACAAAGTAGAGGGCTGCTCGAGTTATACGACTGTTGCTCCGGACTTTGCAGTATATCTGCTGGAGCTCTAGTACGGATT	2nd cloning fragment: *GAL1*_p_-*PRK*-*CYC1*_t_ expression cassette (IMI229) from pUDE046.
11	RV CYC1t-prk HR2	GGAATCTGTGTAGTATGCCTGGAATGTCTGCCGTGCCATAGCCATGTATGCTGATATGTCGTACCGGCCGCAAATTAAAG	2nd cloning fragment: *GAL1*p-*PRK-CYC1*_t_ expression cassette (IMI229) from pUDE046.
12	FW HR2-cbbQ2-HR3	GACATATCAGCATACATGGCTATGG	3rd cloning fragment: *PGI1*_p_-*cbbQ2-TEF2*t cassette (IMI229).
13	RV HR2-cbbQ2-HR3	GGACACGCTTGACAGAATGTCAAAGG	3rd cloning fragment: *PGI1*_p_-*cbbQ2-TEF2*t cassette (IMI229).
14	FW HR3-cbbO2-HR4	CGTCCGATATGATCTGATTGG	4th cloning fragment: *PGK1*_p_-*cbbO2-ADH1*_t_ cassette (IMI229).
15	RV HR3-cbbO2-HR4	CCTAGAAATCTCGTCGATCTC	4th cloning fragment: *PGK1*_p_-*cbbO2-ADH1*_t_ cassette (IMI229).
16	FW HR4-GroEL-HR5	ATCACTCTTACCAGGCTAGG	5th cloning fragment: *TEF1*_p_-*groEL-ACT1*_t_ cassette (IMI229).
17	RV HR4-GroEL-HR5	CTGGACCTTAATCGTGTGCGCATCCTC	5th cloning fragment: *TEF1*_p_-*groEL-ACT1*_t_ cassette (IMI229).
18	FW HR5-GroES-HR6	CCGTATAGCTTAATAGCCAGCTTTATC	6th cloning fragment: *TPI1*_p_-*groES-PGI1*_t_ cassette (IMI229).
19	RV HR5-GroES-HR6	GCTATGACCATGATTACGCCAAGC	6th cloning fragment: *TPI1*_p_-*groES-PGI1*_t_ cassette (IMI229).
20	FW HR6-LEU2-CAN1dwn	CCAGCTTTTGTTCCCTTTAGTGAGGGTTAATTGCGCGCTTGGCGTAATCATGGTCATAGCCTGTGAAGATCCCAGCAAAG	7th (IMI229) or 2nd (IMI232) cloning fragment: *KlLEU2* cassette from pUG73.
21	RV LEU2 HR-CAN1	AGCTCATTGATCCCTTAAACTTTCTTTTCGGTGTATGACTTATGAGGGTGAGAATGCGAAATGGCGTGGAAATGTGATCAAAGGTAATAAAACGTCATATATCCGCAGGCTAACCGGAAC	7th (IMI229) or 2nd (IMI232) cloning fragment: *KlLEU2* cassette from pUG73.
	**Primers used for verification of the *in vivo *assembled constructs**		
22	m-PCR-HR1-FW	GGCGATTAAGTTGGGTAACG	Diagnostic for assembly of plasmids pUDC075, pUDC099, and pUDC100, and integration in strain IMI229.
23	m-PCR-HR1-RV	AACTGAGCTCCAGCTGTACC	Diagnostic for assembly of plasmids pUDC075, pUDC099, pUDC100, and integration in strain IMI229.
24	m-PCR-HR2-FW	ACGCGTGTACGCATGTAAC	Diagnostic for assembly of pUDC075, pUDC099, pUDC100, and integration in strain IMI229
25	m-PCR-HR2-RV	GCGCGTGGCTTCCTATAATC	Diagnostic for assembly of pUDC075, pUDC099, pUDC100, and integration in strain IMI229
26	m-PCR-HR3-FW	GTGAATGCTGGTCGCTATAC	Diagnostic for assembly of pUDC075, pUDC099, pUDC100, and integration in strain IMI229.
27	m-PCR-HR3-RV	GTAAGCAGCAACACCTTCAG	Diagnostic for assembly of pUDC075, pUDC099, pUDC100, and integration in strain IMI229.
28	m-PCR-HR4-FW	ACCTGACCTACAGGAAAGAG	Diagnostic for assembly of pUDC075, pUDC099, pUDC100, and integration in strain IMI229.
29	m-PCR-HR4-RV	TGAAGTGGTACGGCGATGC	Diagnostic for assembly of pUDC075, pUDC099, pUDC100, and integration in strain IMI229.
30	m-PCR-HR5-FW	ATAGCCACCCAAGGCATTTC	Diagnostic for assembly of pUDC075, pUDC099, pUDC100, and integration in strain IMI229.
31	m-PCR-HR5-RV	CCGCACTTTCTCCATGAGG	Diagnostic for assembly of pUDC075, pUDC099, pUDC100, and integration in strain IMI229.
32	m-PCR-HR6-FW	CGACGGTTACGGTGTTAAG	Diagnostic for assembly of pUDC075, pUDC099, pUDC100, and integration in strain IMI229.
33	m-PCR-HR6-RV	CTTCCGGCTCCTATGTTGTG	Diagnostic for assembly of pUDC075, pUDC099, pUDC100, and integration in strain IMI229.

**Table 3:** Plasmids used in this study

**Name**	**Relevant genotype**	**Source/reference**
pFL451	*AOX1*_p_-*prk* (Spinach)-*AOX1*_t_ (pHIL2-D2 *HIS4* Amp centromeric)	Brandes *et al.*[9].
pCR®-Blunt II-TOPO	*Bla*	Life Technologies Europe BV
pTEF424_TEF	*TRP1* 2μ *bla*	Mumberg *et al.*[28].
pSH47	*URA3 CEN6 ARS4 GAL1*_p_-*cre*-*CYC1*_t_*bla*	Güldener *et al*. [29].
pUD0E46	*TRP1* 2μ *GAL1*p-prk-*CYC1*_t_*bla*	This study.
pPCR-Script	*Bla*	Life Technologies Europe BV.
pGPD_426	*URA3* 2μ *bla*	Mumberg *et al.*[28].
pRS416	*URA3 CEN6 ARS4 bla*	Mumberg *et al.*[28].
pBTWW002	*URA3* 2μ *TDH3*_p_-*cbbM-CYC1*_t_*bla*	This study.
pUDC098	*URA3 CEN6 ARS4 TDH3*_p_*-cbbM-CYC1*_t_*bla*	This study.
pMK-RQ	*nptII*	Life Technologies Europe BV.
pUD230	*PGI*1_p_-*cbbQ2*-*TEF2*_t_*nptII*	Life Technologies Europe BV.
pUD231	*PGK1*_p_-*cbbO2-ADH1*_t_*nptII*	Life Technologies Europe BV.
pUD232	*TEF1*_p_-*groEL-ACT1*_t_*nptII*	Life Technologies Europe BV.
pUD233	*TPI1*_p_-*groES*-*PGI1*_t_*nptII*	Life Technologies Europe BV.
pUDC075	*URA3 CEN6 ARS4 TDH3*_p_-*cbbM*-*CYC1*_t;_*PGI1*_p_-*cbbQ2-TEF2*_t_;*PGK1*_p_-*cbbO2-ADH1*_t_;*TEF1*_p_-*groEL-ACT1*_t_*;TPI1*_p_-*groES*-*PGI1*_t_*bla*	This study.
pUDC099	*URA3 CEN6 ARS4 TDH3*_p_-*cbbM*-*CYC1*_t;_*PGI1*_p_-*cbbQ2-TEF2*_t_;*PGK1*_p_-*cbbO2-ADH1*_t_*bla*	This study.
pUDC100	*URA3 CEN6 ARS4 TDH3*_p_-*cbbM*-*CYC1*_t;_*TEF1*_p_-*groEL-ACT1*_t_*;TPI1*_p_-*groES*-*PGI1*_t_*bla*	This study.

Rubisco form II gene *cbbM* from *T. denitrificans*[17] flanked by KpnI and SacI sites was codon optimized [30] (accession number: KC699554), synthesized at GeneArt (Life Technologies Europe BV), and ligated into pPCR-Script. The *cbbM*-containing fragment was ligated into the BamHI and SacI restricted vector pGPD_426 [28] creating plasmid pBTWW002. The *cbbM* expression cassette was transferred into pRS416 using KpnI and SacI, yielding pUDC098.

Expression cassette of the specific Rubisco form II chaperones from *T. denitrificans cbbQ2* and *cbbO2*[20], and chaperones *groEL* and *groES*[18] from *E. coli* were codon optimized [30] (accession numbers: KC699555 and KC699556, respectively). The expression cassettes contained a yeast constitutive promoters and terminator, flanking the codon optimized gene. The cassette was flanked by unique 60-bp regions obtained by randomly combining bar-code sequences used in the *Saccharomyces* Genome Deletion Project [31] and an EcoRV site (GeneArt). The expression cassettes were inserted in plasmid pMK-RQ (GeneArt) using the SfiI cloning sites yielding pUD230 (*PGI1*_p_-*cbbQ2*-*TEF2*_t_), pUD231 (*PGK1*_p_-*cbbO2*-*ADH1*_t_), pUD232 (*TEF1*_p_-*groEL*-*ACT1*_t_), and pUDE233 (*TPI1*_p_-*groES*-*PGI1*_t_) (Table 3). The expression cassette *TDH3*_p_-*cbbM*-*CYC1*_t_ was PCR-amplified from plasmid pBTWW002 using Phusion Hot-Start Polymerase (Finnzymes) and primers HR-cbbM-FW-65 and HR-cbbM-RV-65 in order to incorporate the 60-bp region for recombination cloning.

### Strain construction, isolation and maintenance

All *Saccharomyces cerevisiae* strains used (Table 4) belong to the CEN.PK family [32,33]. All strains were grown in 2% w/v glucose synthetic media [7] supplemented with 150 mg l^-1^ uracil when required [34] until they reached end exponential phase, then sterile glycerol was added up to ca. 30% v/v and aliquot of 1 ml were stocked −80°C.

**Table 4:** ***Saccharomyces cerevisiae *strains used in this study**

	**Relevant genotype**	**Source/reference**
CEN.PK113-5D	*MATa ura3-52*	Euroscarf.
CEN.PK102-3A	*MATa ura3-52 leu2-3, 112*	Euroscarf.
IMC014	*MATa ura3-52* pUDC075 (*CEN6 ARS4 URA3 TDH3*_p_-*cbbM-CYC1*_t_*PGI1*_p_*-cbbQ2-TEF2*_t_*PGK1*_p_*-cbbO2-ADH1*_t_*TEF1*_p_*-groEL-ACT1*_t_*TPI1*_p_*-groES-PGI1*_t_)	This study.
IMC033	*MATa ura3-52* pUDC098 (*CEN6 ARS4 URA3 TDH3*_p_-*cbbM-CYC1*_t_)	This study.
IMC034	*MATa ura3-52* pUDC099 (*CEN6 ARS4 URA3 TDH3*_p_-*cbbM-CYC1*_t_*PGI1*_p_*-cbbQ2-TEF2*_t_*PGK1*_p_*-cbbO2-ADH1*_t_*cbbO2*-pRS416 linker)	This study.
IMC035	*MATa ura3-52* pUDC100 (*CEN6 ARS4 URA3 TEF1*_p_*-groEL-ACT1*_t_*TPI1*_p_*-groES-PGI1*_t_*cbbM*-*GroEL* linker)	This study.
IMI229	*MATa ura3-52 leu2-3, 112 can1Δ::GAL1*_p_*-prk-CYC1*_t_*PGI1*_p_*-cbbQ2-TEF2*_t_*,PGK1*_p_*-cbbO2-ADH1*_t_*,TEF1*_p_*-groEL-ACT1*_t_*,TPI1*_p_*-groES-PGI1*_t_*KlLEU2*	This study.
IMI232	*MATa ura3-52 leu2-3, 112 can1::KlLEU2*	This study.
IMU032	IMI232 p426_GPD (2μ *URA3*)	This study.
IMU033	IMI229 pUDC100 (*CEN6 ARS4 URA3 TEF1*_p_*-groEL-ACT1*_t_*TPI1*_p_*-groES-PGI1*_t_*cbbM*-*GroEL* linker)	This study.

**Figure 4:**
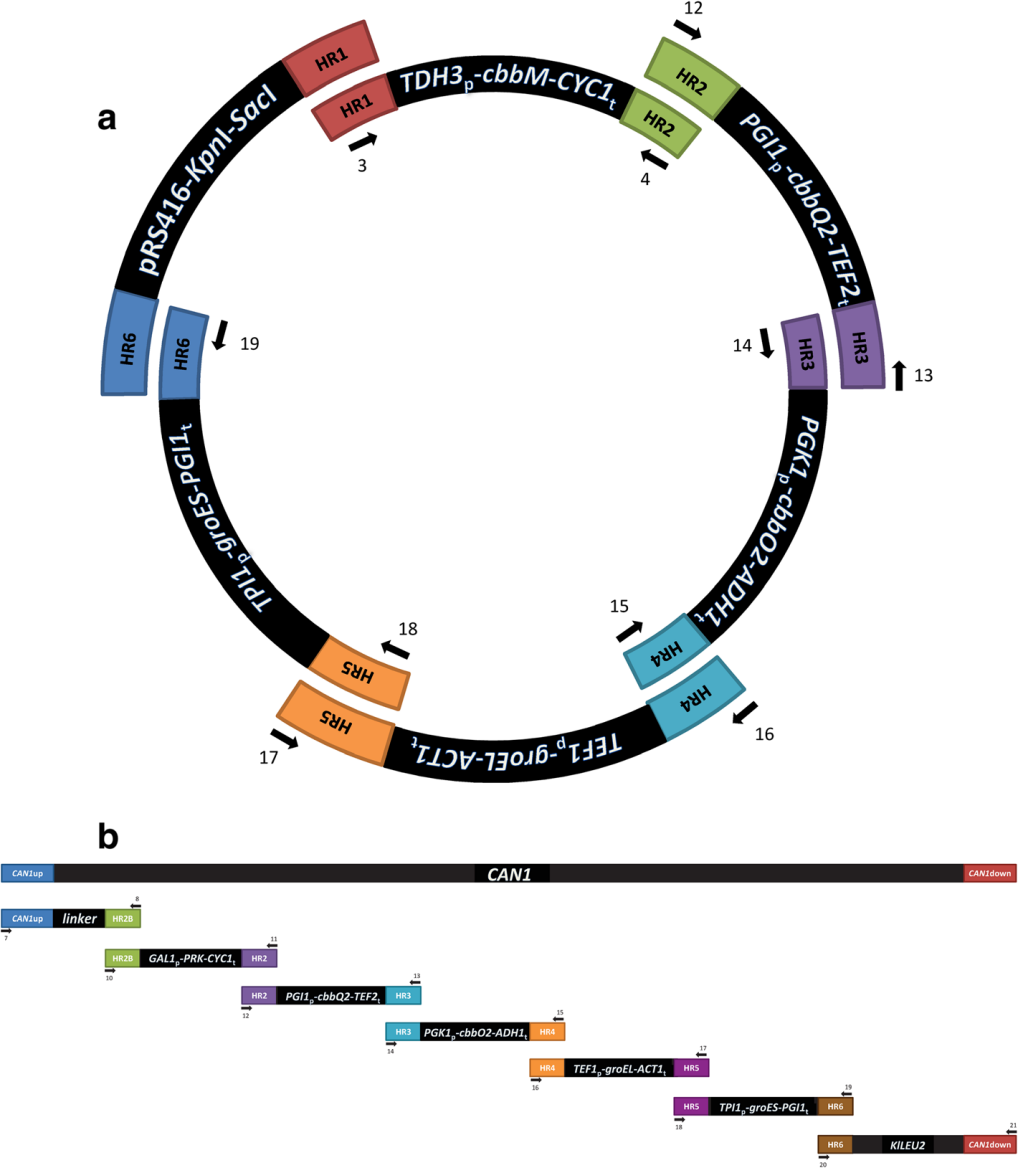
**Strategy for the heterologous expression of Rubisco and PRK in *S. cerevisiae*. (a)***In vivo* assembly of Rubisco expression plasmid pUDC075, and **(b)***in vivo* assembly and integration of PRK and chaperone proteins in *CAN1* locus of *Saccharomyces cerevisiae* strain IMI229. Each fragment represents a different expression cassette or plasmid backbone. All fragments used in assembly experiments were flanked by 60-bp sequences used for *in vivo* recombination, either enabling the assembly of plasmids or the integration assembled constructs into the *S. cerevisiae* genome. Arrows and numbers indicate primers used in the construction of the cassette.

The strain IMC014 that co-expressed the Rubisco form II *cbbM* and the four chaperones *cbbQ2*, *cbbO2*, *groEL*, and *groES* was constructed using a previously published *in vivo* transformation associated recombination [35]. 200 fmol of each expression cassette were pooled with 100 fmol of the KpnI/SacI linearized pRS416 backbone in a final volume of 50 μl and transformed in CEN.PK 113-5D using the lithium acetate protocol [36] (Figure 4a). Cells were selected on synthetic medium. Correct assembly of the fragment of pUDC075 was performed by multiplex PCR on transformant colonies using primers enabling amplification over the regions used for homologous recombination (Table 2) and by restriction analysis after retransformation of the isolated plasmid in *E. coli* DH5α. pUDC075 was sequenced by Next Gen Seq Illumina (100-bp reads paired-end, 50 Mb) and assembled with Velvet [37]. The assembled sequence did not contain mutations in any of the assembled expression cassettes. The strains IMC034 and IMC035 that expressed *cbbM/cbbQ2/cbbO2* and *cbbM/groEL/groES* respectively were constructed using the same *in vivo* assembly method with the following modification. To construct plasmids pUDC099 and pUDC100, 120 bp cbbO2-pRS416 linker and cbbM-GroEL linker were used to close the assembly respectively (Table 2), 100 fmol of each of complementary 120 bp oligonucleotides were added to the transformation. The strain IMC033 that only expressed the *cbbM* gene was constructed by transforming CEN.PK113-5D with pUDC098.

To construct the strain IMU033 that co-expressed PRK, *cbbM*, *cbbQ2*, *cbbO2*, the intermediate strain IMI229 was constructed by integrating PRK, the four chaperones and *KlLEU2*[38] at the *CAN1* locus by *in vivo* homologous integration in CEN.PK102-3A (Figure 4b). The expression cassettes were PCR amplified using Phusion Hot-Start Polymerase (Finnzymes), the corresponding oligonucleotides and DNA templates (Table 2). Finally, the strain IMI229 was transformed with pUDC100 that carries the Rubisco form II *cbbM* and the two *E. coli* chaperones *groEL* and *groES*.

Strain IMI232 was constructed by transforming CEN.PK102-3A with the *KlLEU2* cassette. IMI232 was finally transformed with the plasmid p426GPD to restore prototrophy resulting in the reference strain IMU032.

### Experimental set-up of chemostat and batch experiments

Anaerobic chemostat cultivation was performed essentially as described [39] but with 12.5 g l^-1^ glucose and 12.5 g l^-1^ galactose as the carbon source and where indicated, a mixture of 10% CO_2_/90% N_2_ replaced pure nitrogen as the sparging gas. Residual glucose and galactose concentrations were determined after rapid quenching [40] using commercial enzymatic assays for glucose (Boehringer, Mannheim, Germany) and D-galactose (Megazyme, Bray, Ireland). Anaerobic bioreactor batch cultures were grown essentially as described [6], but with 20 g l^-1^ galactose and a sparging gas consisting of 10% CO_2_ and 90% N_2_. Biomass and metabolite concentrations in batch and chemostat and batch cultures were determined as described by Guadalupe *et al.*[6]. In calculations of ethanol fluxes and yields, ethanol evaporation was corrected for based on a first-order evaporation rate constant of 0.008 h^-1^ in the bioreactor setups and under the conditions used in this study [6,39].

### Enzyme assays for phosphoribulokinase and Rubisco

Cell extracts for analysis of phosphoribulokinase (PRK) activity were prepared as described previously [41]. PRK activity was measured at 30°C by a coupled spectrophotometric assay [42]. Reaction rates were proportional to the amounts of cell extract added. Protein concentrations were determined by the Lowry method [43] using bovine serum albumin as a standard.

Cell extracts for Rubisco activity assays were prepared as described [41], with two modifications: Tris–HCl (1 mM, pH 8.2) containing 20 mM MgCl_2_•6H_2_O, 5 mM of DTT and 5 mM NaHCO_3_ was used as sonication buffer and Tris–HCl (100 mM, pH 8.2), 20 mM MgCl_2_•6H_2_O and 5 mM of DTT as freezing buffer. Rubisco activity was determined by measuring ^14^CO_2_-fixation (PerkinElmer, Groningen, The Netherlands) as described [44] and measuring radioactive counts in a TRI-CARB® 2700TR Series liquid scintillation counter (PerkinElmer, Groningen, The Netherlands), using Ultima Gold™ scintillation cocktail (PerkinElmer, Groningen, The Netherlands). Protein concentrations were determined by the Lowry method [43] using standard solutions of bovine serum albumin dissolved in 50 mM Tris–HCl (pH 8.2).

## Abbreviations

Rubisco: Ribulose 1,5-bisphosphate carboxylase/oxidase; PRK: Phosphoribulokinase; NAD+: Nicotinamide adenine dinucleotide; NADP+: Nicotinamide adenine dinucleotide phosphate.

## Competing interests

VGM, HWW, AJAvM, and JTP are inventors on a patent application related to the content of this article.

## Authors’ contributions

VGM, JMD, JTP, AJAvM designed the experiments and wrote the manuscript. HWW selected the Rubisco and PRK genes, VGM selected the chaperone genes and constructed all yeast strains. VGM and EdH carried out and analyzed the fermentation experiments. VGM and MAHL performed the PRK and Rubisco enzymatic activity determinations. All the authors have read and approved the final manuscript.
